# Supplementary figures and images for: Deep mutational scanning reveals the functional constraints and evolutionary potential of the influenza A virus PB1 protein
Source: J Virol. 2023 Oct 26;97(11):e01329-23. doi: 10.1128/jvi.01329-23 (PMC10688322; doi:10.1128/jvi.01329-23)

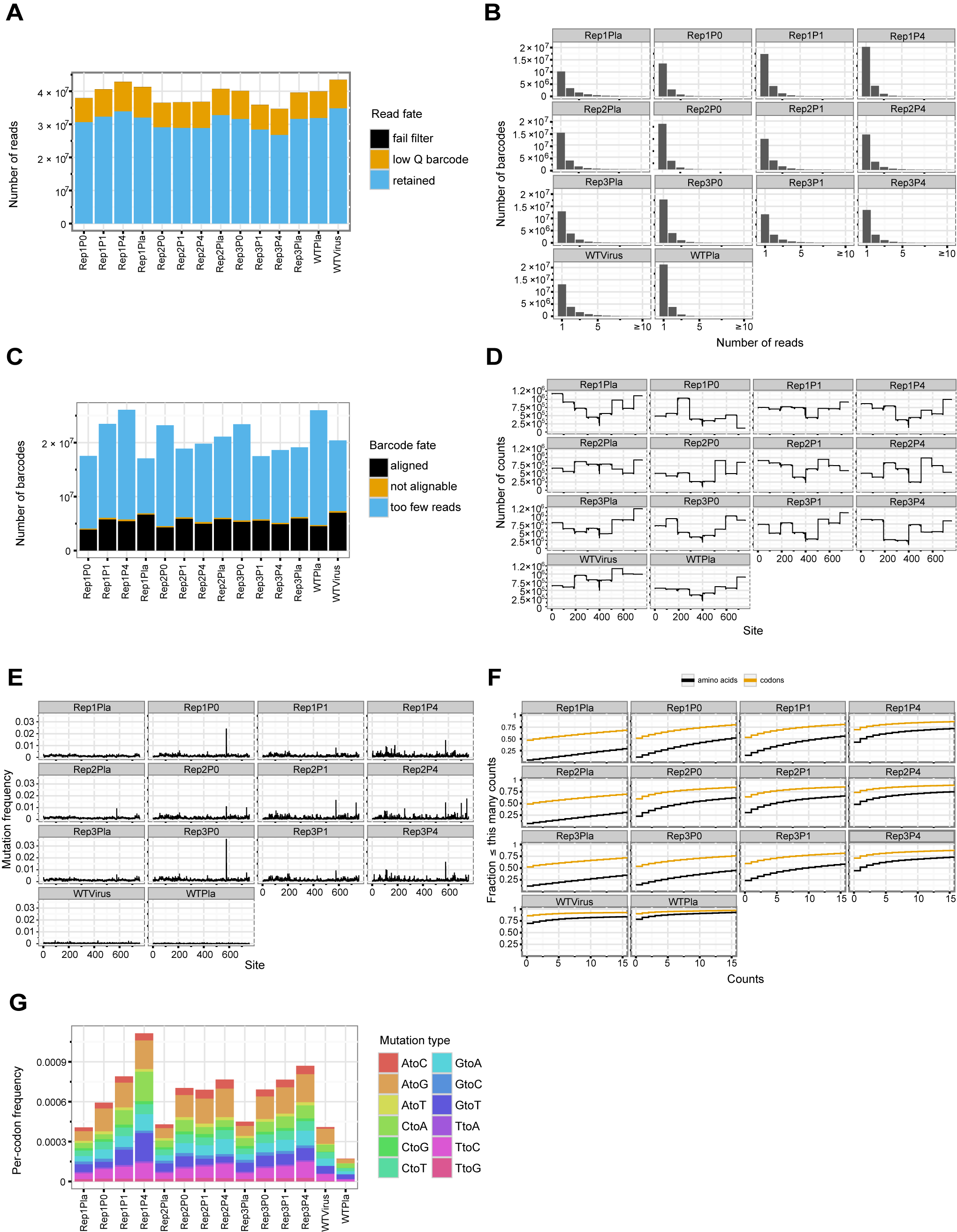

Supplement: Figure S1 — Full description of deep mutational scanning libraries. [file jvi.01329-23-s0001.tif]

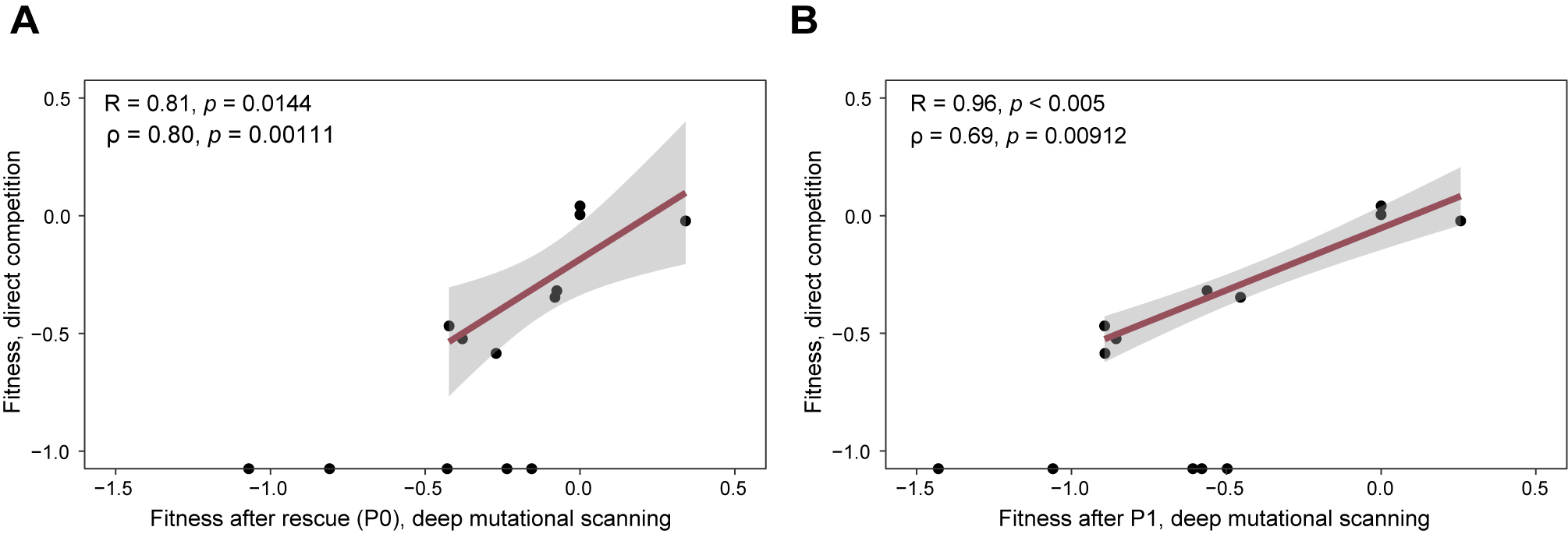

Supplement: Figure S2 — Fitness comparison between deep mutational scanning and direct competition in early passages. [file jvi.01329-23-s0002.tif]

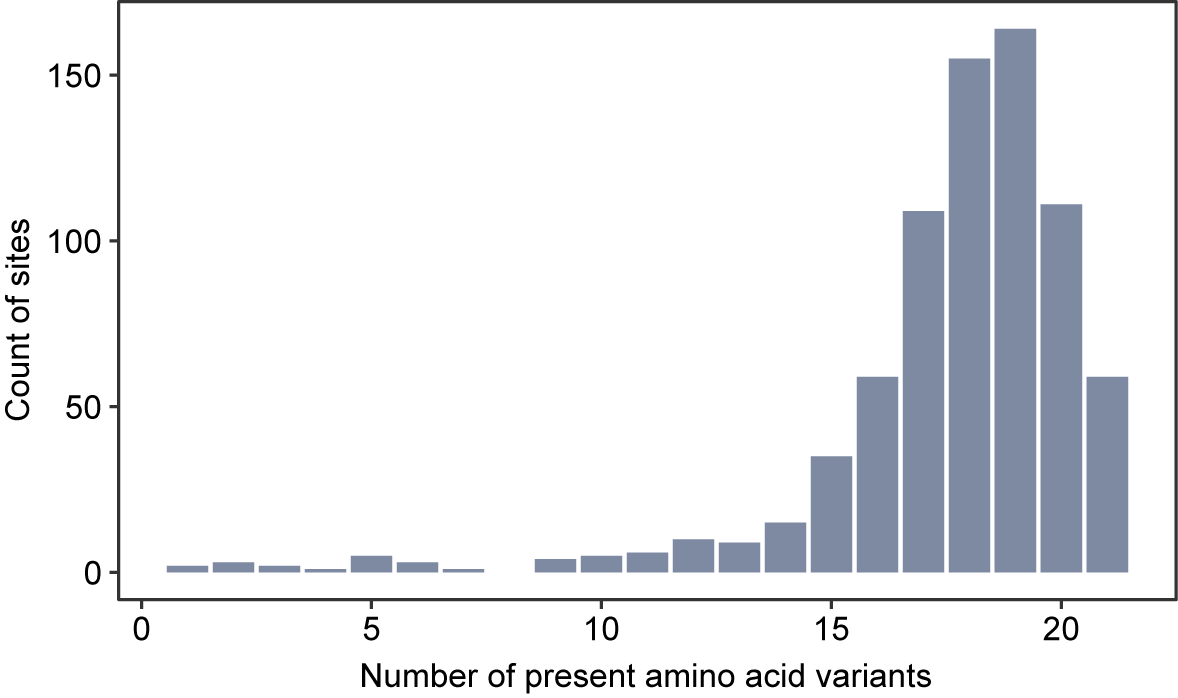

Supplement: Figure S3 — Sites with varying mutational representation. [file jvi.01329-23-s0003.tif]

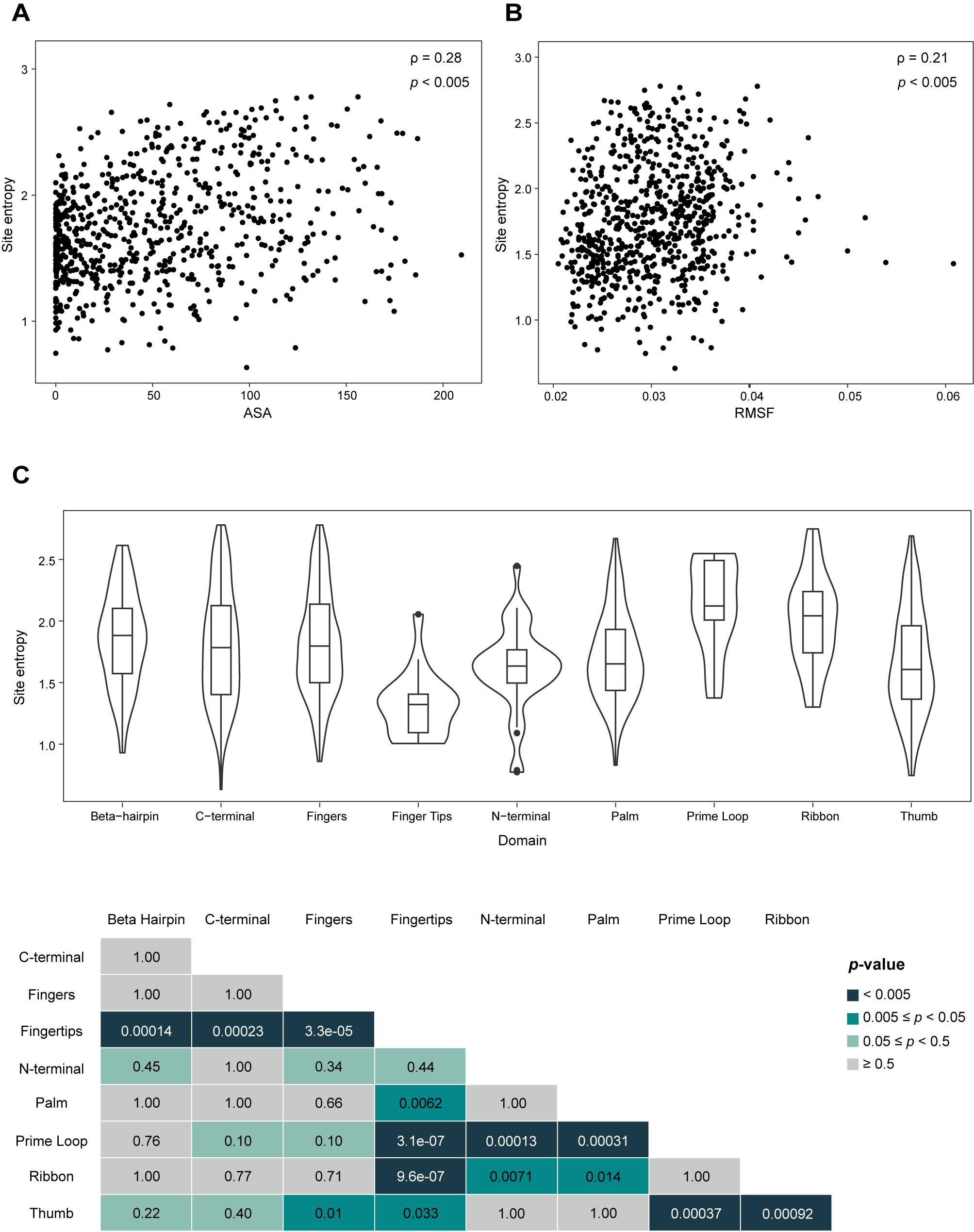

Supplement: Figure S4 — Correlation between site entropy and defined features on RdRp. [file jvi.01329-23-s0004.tif]

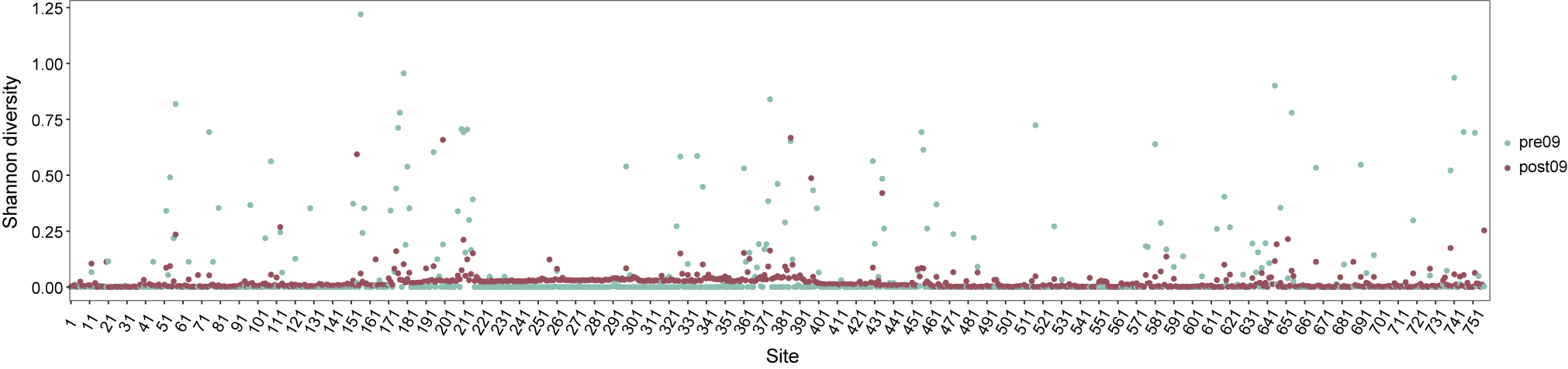

Supplement: Figure S5 — Amino acid diversity at sites of naturally occurring influenza H1N1 PB1 sequences. [file jvi.01329-23-s0005.tif]

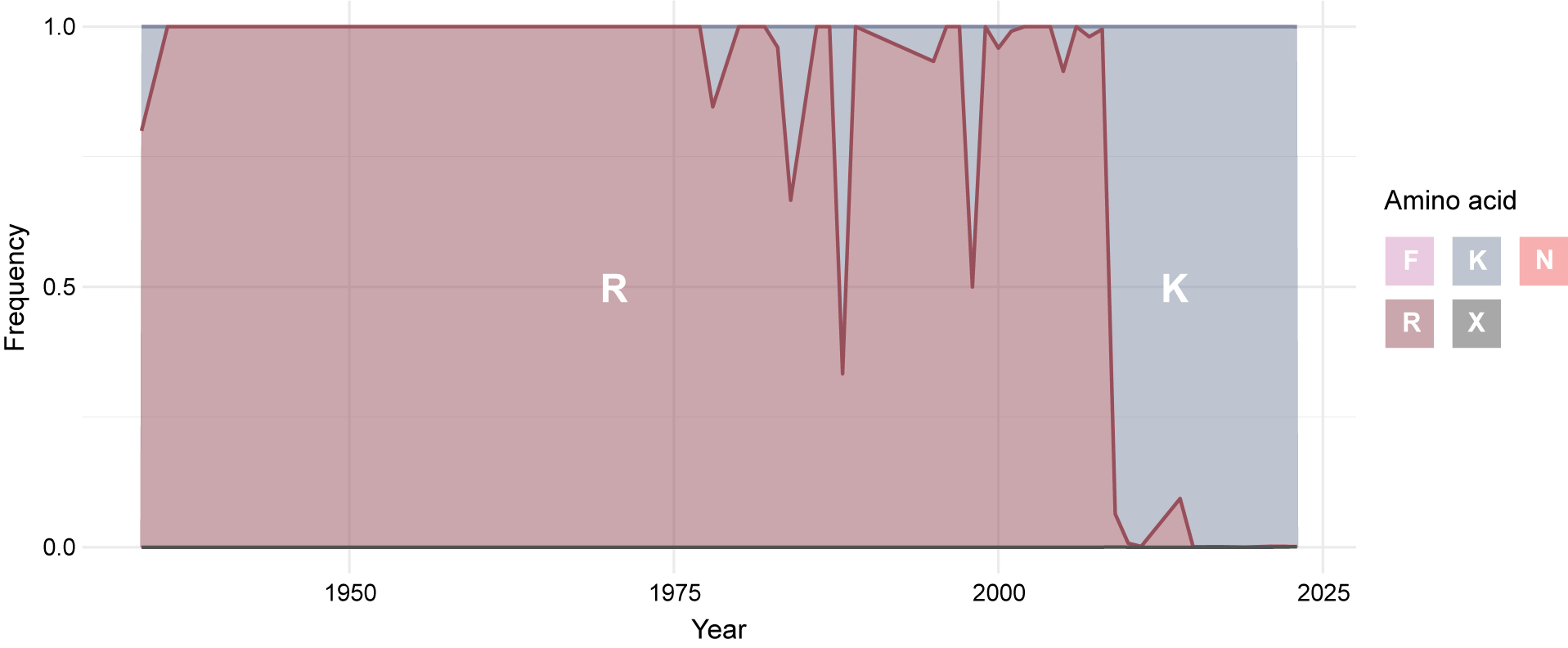

Supplement: Figure S6 — Frequency change of amino acid variants at site 691. [file jvi.01329-23-s0006.tif]

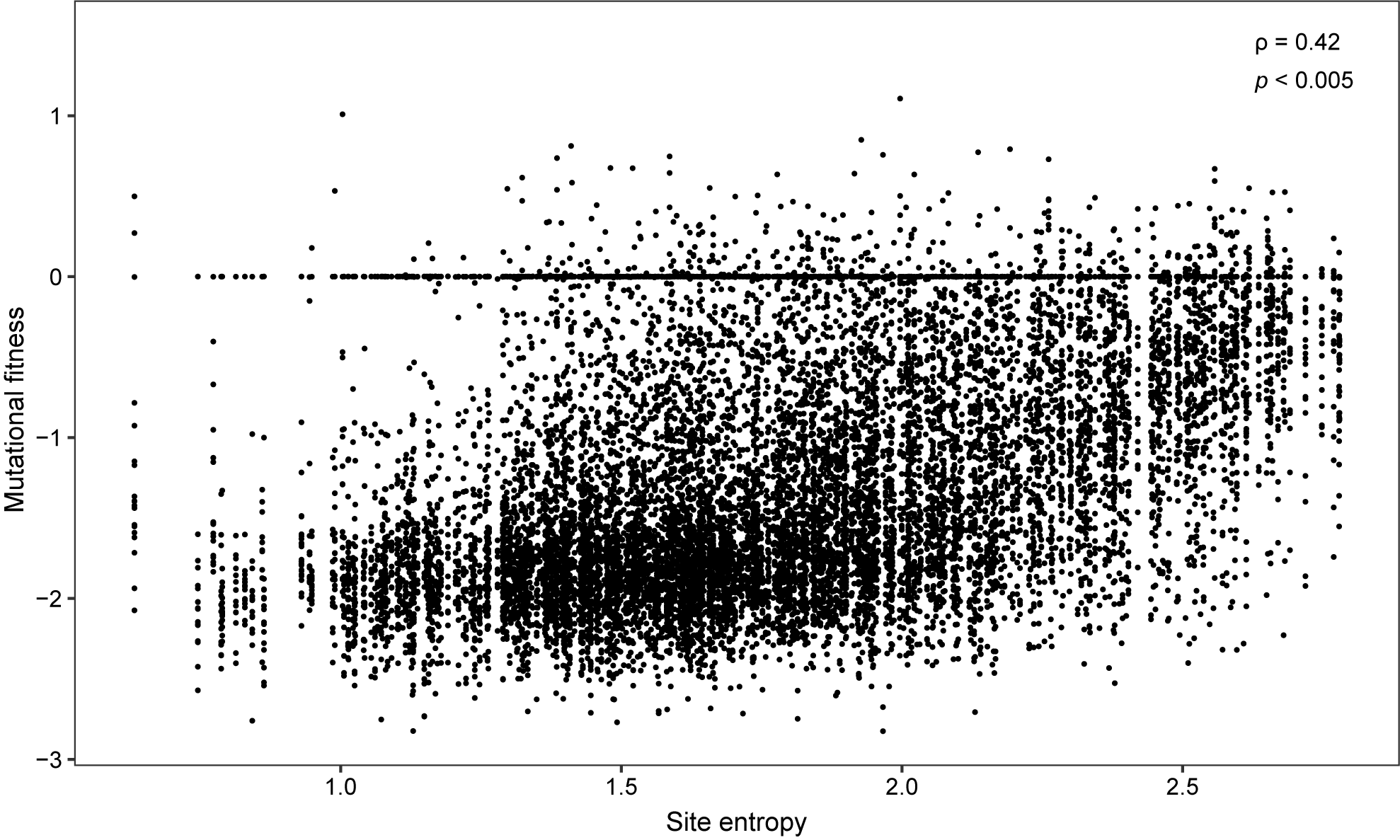

Supplement: Figure S7 — Correlation between site entropy and mutational fitness. [file jvi.01329-23-s0007.tif]
