# Supplementary material for: Deep mutational scanning reveals the functional constraints and evolutionary potential of the influenza A virus PB1 protein
Source: J Virol. 2023 Oct 26;97(11):e01329-23. doi: 10.1128/jvi.01329-23 (PMC10688322; doi:10.1128/jvi.01329-23)
Supplement: Supplemental text — Supplemental figure legends, primers, PCR conditions. [file jvi.01329-23-s0009.docx]

**Supplemental Figure Legends**

**Supplemental Figure 1. Full description of deep mutational scanning libraries.** (A) Raw sequencing reads of each sample. The “filter” in “fail filter” refers to general Illumina filters. “Low Q barcode” refers to sequences having any nucleotide with a Q-score below 15 in the 16× N molecule-specific barcodes. Sequences that failed the filter or with low-Q barcodes were discarded in subsequent analyses. (B) The number of distinct barcodes observed in each sample. Each barcode needs to be observed at least twice to determine the consensus sequence for that contig. The bar at 1 corresponds either to barcodes that were only observed once or to sequencing errors that gave rise to new barcodes. (C) Barcodes after aligning to wild type WSN33 PB1 sequence. “Too few reads” corresponds to the bar at 1 in panel (B). Sequences categorized as “too few reads” were removed from subsequent analyses. (D) Sequencing depth at each site in PB1 after removing contigs with too few reads. The number of counts includes the codon counts for both variant and wild type codons. (E) The mutational frequency at each site in PB1 after removing contigs with too few reads. The spike at site 577 in library Rep1P0, Rep1P4, Rep3P0, and Rep3P4 is likely an issue with the sequencing library preparation, as other sequencing runs using the same samples did not show such peaks (data not shown). The spikes have little impact on the type of codons present in the libraries. The impact of peaks on fitness measurements is also minimal since we compared the passaged libraries to the plasmid libraries. (F) Mutation sampling completeness. The plot shows the fraction of codon and amino acid mutations observed no more than the indicated number of times. This plot describes both variant diversity and sequencing completeness in a library. (G) Frequency of different types of nucleotide change. The plot shows nucleotide change among mutations with only one nucleotide change and works as a check for oxidative damage. An excessive number of C to A or G to T mutations suggests potential oxidative damage. The plot shows no over-representation of either mutation in the libraries.

**Supplemental Figure 2. Fitness comparison between deep mutational scanning and direct competition in early passages.** The comparison between the replicative fitness measured by direct competition with the wild type strain and by deep mutational scanning (A) After virus library rescue, before passaging, and (B) after one passage on A549 cells. R indicates the Pearson correlation coefficient for viable variants, while ρ indicates the Spearman correlation coefficient for all variants including lethal mutations. The red line shows the trendline using a linear regression model. The gray zone indicates the 95% confidence interval for predictions from the linear model.

**Supplemental Figure 3. Sites with varying mutational representation.** Count of sites with different numbers of amino acid variants present at that site in the plasmid libraries, after filtering out the mutations with low sequencing counts or with sequencing library preparation errors. The maximum number includes twenty amino acid variants plus stop codons at each site.

**Supplemental Figure 4. Correlation between site entropy and defined features on RdRp.** Correlation between a residue’s (A) accessible surface area or (B) root mean square fluctuation, and its site entropy. Each dot represents a residue on the RdRp. ρ indicates the Spearman correlation coefficient. (C) Site entropy distribution in different subdomains of RdRp. The chart below shows the adjusted p-values by Bonferroni correction between each pair of subdomain comparisons.

**Supplemental Figure 5. Amino acid diversity at sites of naturally occurring influenza H1N1 PB1 sequences.** Weighted Shannon diversity for each site in natural PB1 evolution. Diversity in pre- and post-2009 sequences was calculated separately.

**Supplemental Figure 6. Frequency change of amino acid variants at site 691.** Frequency of amino acid variants observed at site 691 of naturally occurring PB1 sequences from 1934 to 2023. “X” stands for uncertain/ambiguous amino acid. Dominant amino acid variants were labeled on the plot.

**Supplemental Figure 7. Correlation between site entropy and mutational fitness.** Each dot represents an amino acid substitution at a site. ρ indicates the Spearman correlation coefficient.

**Primers and PCR Conditions**

**PCR0**

| Rnd0_Fwd | AGCGAAAGCAGGCAAACCATTTGA |
| --- | --- |
| Rnd0_Rev | GGCATTTTTTCATGAAGGACAAGCTAAATTCA |

**PCR1**

| Rnd1_Fwd1 | CTTTCCCTACACGACGCTCTTCCGATCTNNNNNNNNAGCGAAAGCAGGCAAACCATTTGA |
| --- | --- |
| Rnd1_Rev1 | GGAGTTCAGACGTGTGCTCTTCCGATCTNNNNNNNNACCAGGATGGGATTCCTCAAGGAA |
| Rnd1_Fwd2 | CTTTCCCTACACGACGCTCTTCCGATCTNNNNNNNNGATTGTGTATTGGAAGCAATGGCC |
| Rnd1_Rev2 | GGAGTTCAGACGTGTGCTCTTCCGATCTNNNNNNNNCTTAGTCATATTGTCTCTCACTCG |
| Rnd1_Fwd3 | CTTTCCCTACACGACGCTCTTCCGATCTNNNNNNNNGAGATCACAACTCATTTTCAGAGAAAGAGA |
| Rnd1_Rev3 | GGAGTTCAGACGTGTGCTCTTCCGATCTNNNNNNNNGAAATTTCAGTGTCCTGAGAATTGGT |
| Rnd1_Fwd4 | CTTTCCCTACACGACGCTCTTCCGATCTNNNNNNNNGGCAAATGTTGTAAGGAAGATGATG |
| Rnd1_Rev4 | GGAGTTCAGACGTGTGCTCTTCCGATCTNNNNNNNNCATTCCAGGGCTCAATGATGC |
| Rnd1_Fwd5 | CTTTCCCTACACGACGCTCTTCCGATCTNNNNNNNNCGGCCGCTCTTAATAGATGGGACT |
| Rnd1_Rev5 | GGAGTTCAGACGTGTGCTCTTCCGATCTNNNNNNNNGCTGGGAAGCTCCATGCTGAAATT |
| Rnd1_Fwd6 | CTTTCCCTACACGACGCTCTTCCGATCTNNNNNNNNTTCTATCGTTATGGGTTTGTTGCC |
| Rnd1_Rev6 | GGAGTTCAGACGTGTGCTCTTCCGATCTNNNNNNNNGTATAAATTTGGGCCTCCGTC |
| Rnd1_Fwd7 | CTTTCCCTACACGACGCTCTTCCGATCTNNNNNNNNAAAGCTGGACTGCTGGTCTCC |
| Rnd1_Rev7 | GGAGTTCAGACGTGTGCTCTTCCGATCTNNNNNNNNGTTGCAGCACTTTTGGTACATTTG |
| Rnd1_Fwd8 | CTTTCCCTACACGACGCTCTTCCGATCTNNNNNNNNGCCAAAGAGGAATACTTGAAGATGAA |
| Rnd1_Rev8 | GGAGTTCAGACGTGTGCTCTTCCGATCTNNNNNNNNGGCATTTTTTCATGAAGGACAAGCTAAATTCA |

**PCR2**

| Rnd2_Fwd1 | AATGATACGGCGACCACCGAGATCTACACTCGTGGAGCGACACTCTTTCCCTACACGACGCTCTTCCGATCT |
| --- | --- |
| Rnd2_Rev1 | CAAGCAGAAGACGGCATACGAGATCGCTCAGTTCGTGACTGGAGTTCAGACGTGTGCTCTTCCGATCT |
| Rnd2_Fwd2 | AATGATACGGCGACCACCGAGATCTACACCTACAAGATAACACTCTTTCCCTACACGACGCTCTTCCGATCT |
| Rnd2_Rev2 | CAAGCAGAAGACGGCATACGAGATTATCTGACCTGTGACTGGAGTTCAGACGTGTGCTCTTCCGATCT |
| Rnd2_Fwd3 | AATGATACGGCGACCACCGAGATCTACACTATAGTAGCTACACTCTTTCCCTACACGACGCTCTTCCGATCT |
| Rnd2_Rev3 | CAAGCAGAAGACGGCATACGAGATATATGAGACGGTGACTGGAGTTCAGACGTGTGCTCTTCCGATCT |
| Rnd2_Fwd4 | AATGATACGGCGACCACCGAGATCTACACACCAGCGACAACACTCTTTCCCTACACGACGCTCTTCCGATCT |
| Rnd2_Rev4 | CAAGCAGAAGACGGCATACGAGATTCGTCTGACTGTGACTGGAGTTCAGACGTGTGCTCTTCCGATCT |
| Rnd2_Fwd5 | AATGATACGGCGACCACCGAGATCTACACCATACACTGTACACTCTTTCCCTACACGACGCTCTTCCGATCT |
| Rnd2_Rev5 | CAAGCAGAAGACGGCATACGAGATGAACATACGGGTGACTGGAGTTCAGACGTGTGCTCTTCCGATCT |
| Rnd2_Fwd6 | AATGATACGGCGACCACCGAGATCTACACTCGGCAGCAAACACTCTTTCCCTACACGACGCTCTTCCGATCT |
| Rnd2_Rev6 | CAAGCAGAAGACGGCATACGAGATAACCATTCTCGTGACTGGAGTTCAGACGTGTGCTCTTCCGATCT |
| Rnd2_Fwd7 | AATGATACGGCGACCACCGAGATCTACACCTAATGATGGACACTCTTTCCCTACACGACGCTCTTCCGATCT |
| Rnd2_Rev7 | CAAGCAGAAGACGGCATACGAGATGGTTGCCTCTGTGACTGGAGTTCAGACGTGTGCTCTTCCGATCT |
| Rnd2_Fwd8 | AATGATACGGCGACCACCGAGATCTACACGGTTGCCTCTACACTCTTTCCCTACACGACGCTCTTCCGATCT |
| Rnd2_Rev8 | CAAGCAGAAGACGGCATACGAGATCTAATGATGGGTGACTGGAGTTCAGACGTGTGCTCTTCCGATCT |
| Rnd2_Fwd9 | AATGATACGGCGACCACCGAGATCTACACCGCACATGGCACACTCTTTCCCTACACGACGCTCTTCCGATCT |
| Rnd2_Rev9 | CAAGCAGAAGACGGCATACGAGATTCGGCCTATCGTGACTGGAGTTCAGACGTGTGCTCTTCCGATCT |
| Rnd2_Fwd10 | AATGATACGGCGACCACCGAGATCTACACGGCGAGATGGACACTCTTTCCCTACACGACGCTCTTCCGATCT |
| Rnd2_Rev10 | CAAGCAGAAGACGGCATACGAGATTTCTATGGTTGTGACTGGAGTTCAGACGTGTGCTCTTCCGATCT |
| Rnd2_Fwd11 | AATGATACGGCGACCACCGAGATCTACACAATAGAGCAAACACTCTTTCCCTACACGACGCTCTTCCGATCT |
| Rnd2_Rev11 | CAAGCAGAAGACGGCATACGAGATCCTCGCAACCGTGACTGGAGTTCAGACGTGTGCTCTTCCGATCT |
| Rnd2_Fwd12 | AATGATACGGCGACCACCGAGATCTACACTCGTATGCGGACACTCTTTCCCTACACGACGCTCTTCCGATCT |
| Rnd2_Rev12 | CAAGCAGAAGACGGCATACGAGATATGTCGTGGTGTGACTGGAGTTCAGACGTGTGCTCTTCCGATCT |
| Rnd2_Fwd13 | AATGATACGGCGACCACCGAGATCTACACGTCGATTACAACACTCTTTCCCTACACGACGCTCTTCCGATCT |
| Rnd2_Rev13 | CAAGCAGAAGACGGCATACGAGATCGTATAATCAGTGACTGGAGTTCAGACGTGTGCTCTTCCGATCT |
| Rnd2_Fwd14 | AATGATACGGCGACCACCGAGATCTACACAGTGGTCAGGACACTCTTTCCCTACACGACGCTCTTCCGATCT |
| Rnd2_Rev14 | CAAGCAGAAGACGGCATACGAGATGCCATTAGACGTGACTGGAGTTCAGACGTGTGCTCTTCCGATCT |

**Cycling Programs**

**PCR0**

| **Step** | **Temperature(℃)** | **Time(sec)** |
| --- | --- | --- |
| 1. polymerase activation | 95 | 120 |
| 2. denaturing | 95 | 20 |
|  | 70 | 1 |
| 3. annealing | 52 | 30 |
| 4. extension | 70 | 50 |
| Repeat step 2-4 | For 22 cycles in total | |
| 5. hold | 4 | |

**PCR1**

| **Step** | **Temperature(℃)** | **Time(sec)** |
| --- | --- | --- |
| 1. polymerase activation | 95 | 120 |
| 2. denaturing | 95 | 20 |
|  | 70 | 1 |
| 3. annealing | 54 | 20 |
| 4. extension | 70 | 20 |
| Repeat step 2-4 | For 9 cycles in total | |
| 5. termination | 95 | 60 |
| 6. hold | 4 | |

**PCR2**

| **Step** | **Temperature(℃)** | **Time(sec)** |
| --- | --- | --- |
| 1. polymerase activation | 95 | 120 |
| 2. denaturing | 95 | 20 |
|  | 70 | 1 |
| 3. annealing | 55 | 20 |
| 4. extension | 70 | 20 |
| Repeat step 2-4 | For 24 cycles in total | |
| 5. hold | 4 | |
